# The natural anti-tumor compound Celastrol targets a Myb-C/EBPβ-p300 transcriptional module implicated in myeloid gene expression

**DOI:** 10.1371/journal.pone.0190934

**Published:** 2018-02-02

**Authors:** Anna Coulibaly, Astrid Haas, Simone Steinmann, Anke Jakobs, Thomas J. Schmidt, Karl-Heinz Klempnauer

**Affiliations:** 1 Institute for Biochemistry, Westfälische-Wilhelms-Universität, D-48149 Münster, Germany; 2 Institute for Pharmaceutical Biology and Phytochemistry, Westfälische-Wilhelms-Universität, D-48149 Münster, Germany; Emory University, UNITED STATES

## Abstract

Myb is a key regulator of hematopoietic progenitor cell proliferation and differentiation and has emerged as a potential target for the treatment of acute leukemia. Using a myeloid cell line with a stably integrated Myb-inducible reporter gene as a screening tool we have previously identified Celastrol, a natural compound with anti-tumor activity, as a potent Myb inhibitor that disrupts the interaction of Myb with the co-activator p300. We showed that Celastrol inhibits the proliferation of acute myeloid leukemia (AML) cells and prolongs the survival of mice in an in vivo model of AML, demonstrating that targeting Myb with a small-molecule inhibitor is feasible and might have potential as a therapeutic approach against AML. Recently we became aware that the reporter system used for Myb inhibitor screening also responds to inhibition of C/EBPβ, a transcription factor known to cooperate with Myb in myeloid cells. By re-investigating the inhibitory potential of Celastrol we have found that Celastrol also strongly inhibits the activity of C/EBPβ by disrupting its interaction with the Taz2 domain of p300. Together with previous studies our work reveals that Celastrol independently targets Myb and C/EBPβ by disrupting the interaction of both transcription factors with p300. Myb, C/EBPβ and p300 cooperate in myeloid-specific gene expression and, as shown recently, are associated with so-called super-enhancers in AML cells that have been implicated in the maintenance of the leukemia. We hypothesize that the ability of Celastrol to disrupt the activity of a transcriptional Myb-C/EBPβ-p300 module might explain its promising anti-leukemic activity.

## Introduction

The transcription factor Myb plays a key role as a regulator of proliferation and differentiation of hematopoietic progenitor cells and has been implicated in the development of acute leukemia [1]. Genomic rearrangements of the *MYB* gene and mutations that create de-novo Myb binding sites in the transcriptional control region of the *TAL1* oncogene have been detected in acute lymphoid leukemia [2–4]. Furthermore, acute myeloid leukemia (AML) cells are addicted to high levels of Myb expression, which makes them more vulnerable to Myb inhibition than their normal counterparts [5–7]. Recent genome-wide studies have identified super-enhancers, i.e. cis-regulatory regions that are significantly larger than typical enhancers and that drive the expression of genes involved in cell identity and disease [8–10]. In AML cells, super-enhancers are densely loaded with chromatin regulators such as p300 and BRD4 and hematopoietic transcription factors, such as Myb, C/EBPα, C/EBPβ, ERG, FLI1, and PU.1 [11]. These studies have confirmed the important role of Myb and C/EBPs for the proliferation of AML cells, suggesting a mechanistic framework for the addiction of AML cells to high levels of Myb expression. Targeting Myb with small-molecule inhibitors therefore offers potential for the development of novel therapeutic strategies for the treatment of AML and other tumors with deregulated Myb, such as adenoid cystic carcinoma or specific subtypes of glioblastoma [12,13].

In myeloid cells Myb cooperates with members of the C/EBP family, basic-region-leucine-zipper (bzip) transcription factors that are involved in the differentiation and proliferation of various cell types [14]. C/EBP activity is controlled by various post-translational modifications [15–20] and by the expression of alternative isoforms based on translation initiation at distinct start codons [21,22].

We have previously established a myeloid reporter cell line as a screening tool to search for low molecular weight Myb inhibitors [23]. These cells carry a stably integrated Myb-responsive reporter gene driven by the promoter and enhancer of the myeloid-specific chicken *mim-1* gene, a Myb target gene whose expression is strongly induced by Myb in myeloid cells. Using these cells we have identified and characterized several compounds as potential Myb inhibitors [24–27]. One of these compounds, the pentacyclic triterpenoid Celastrol, was shown to inhibit Myb by disrupting the interaction of Myb transactivation domain with the Kix domain of the co-activator p300 [25]. Importantly, we also showed that Celastrol inhibits the proliferation of primary mouse AML cells and leukemic cells from AML patients while the proliferation of normal murine or human early hematopoietic progenitor cells was not affected [25]. Furthermore, in vivo experiments showed that Celastrol significantly prolonged the survival of mice in a model of an aggressive AML [25]. Taken together, these findings demonstrated that targeting of Myb by a small-molecule inhibitor is feasible and might have therapeutic potential for the treatment of AML.

We recently became aware that our cell-based screening assay also responds to inhibition of C/EBPβ, as exemplified by the sesquiterpene lactone helenalin acetate, a compound that inhibits Myb-induced *mim-1* expression but was shown to be a potent inhibitor of C/EBPβ instead of a Myb inhibitor [28,29]. We showed before that Myb requires C/EBP family members, such as C/EBPα or C/EBPβ, as essential cooperation partners to stimulate the *mim-1* gene [30,31], explaining why inhibition of C/EBPβ has a similar effect as the inhibition of Myb in our screening system. We have therefore re-investigated the inhibitory mechanism of Celastrol to know if Celastrol also exerts inhibitory effects on C/EBPβ. Surprisingly, our data show that Celastrol is also a highly potent inhibitor of C/EBPβ suggesting that the promising inhibitory effects of Celastrol on AML cells might be due to its ability to simultaneously target Myb and C/EBPβ.

## Materials and methods

### Cells

The quail fibroblast cell line QT6 was originally obtained from P. Vogt (La Jolla). 3T3-L1 cells were obtained from ATCC 5 year ago and were used within 10 passages from thawing. All cells were tested on a regular basis and were free of mycoplasma contamination. Adipocyte differentiation was induced as published [32]. Celastrol was from Sigma-Aldrich, dissolved in DMSO and stored at 10 mM stock solution at -70°C.

### Transfections

QT6 fibroblasts were transfected by calcium-phosphate co-precipitation, followed by analysis of the transfected cells for mRNA expression, protein interaction or reporter gene activities. A constant amount of the β-galactosidase reporter plasmid pCMVβ was always included, allowing transfection efficiencies to be normalized according to the β-galactosidase activities. Reporter gene activities were analyzed as described [30,33]. The luciferase reporter genes pG5E4-38Luc and p-240Luc contain 5 tandem copies of Gal4-binding sites or the promoter of the chicken *mim-1* gene, respectively. Luciferase values were divided by the β-galactosidase activity to compensate different transfection efficiencies. All reporter studies were performed in at least 3 independent experiments, with replicate transfections in each experiment. Northern blotting was performed as described [30,33]. Blots were hybridized sequentially with radioactive probes corresponding to the C/EBPβ-inducible MRP126 mRNA and the ribosomal protein S17 mRNA (as housekeeping control). Radioactive bands were detected and quantified using a phosphor image analyzer. Electrophoretic mobility shift assays (EMSA) were performed as described [34]. Briefly, a pair of complementary single-stranded oligonucleotides containing a consensus C/EBP binding site was annealed and used for gel retardation assays: 5’-TGTAGCTGCAGATTGCGCAATCTGCATCTA-3’ and 5’-GTAGATGCAGATTGCGCAATCTGCAGCTACA-3’. After annealing, oligonucleotides were radiolabeled by filling-in the ends using α^32^P-dCTP and Klenow polymerase. Nuclear extract from QT6 cells transfected with C/EBPβ expression vector and treated with different concentrations of Celastrol was prepared as described [34].

### Expression vectors and transfections

Expression vectors for chicken and mouse C/EBPβ, a C/EBPβ mutant lacking all cysteine residues (CallA) and chicken C/EBPα have been described [16,29,30,35]. Expression vectors for full-length human p300 and truncated p300 constructs p300/1751-2370 and p300/1751-1947 have been described [33]. Expression vectors for the p300/1751-1947 mutations C1789A, C1790A and C1789,1790A (CC) were generated by oligonucleotide-directed mutagenesis. Plasmids encoding Gal4-C/EBPβ, p300-VP16, Gal4-VP16 and C/EBPβ-YFP fusion proteins were described before [29,33].

### GFP-trap

QT6 fibroblasts expressing YFP or C/EBPβ-YFP were lysed 16 hours post transfection in ELB buffer containing 120mM sodium chloride, 50mM Tris/HCL, pH 7.4, 20mM sodium fluoride, 1mM EDTA, 6mM EGTA, 15mM sodium pyrophosphate, 1mM Phenylmethylsulfonyl Fluoride (PMSF), 0.5% NP-40 and centrifuged for 20 min at 14,000 x g. An aliquot of the supernatant was retained as input control. The remaining supernatant was incubated with GFP-trap beads (Chromotec, München) for 3 hours at 4°C. Beads were washed three times with ELB buffer, boiled in sodium dodecyl sulfate (SDS) sample buffer and analyzed together with the input samples by SDS-PAGE and western blotting, using appropriate antibodies.

## Results

### Celastrol is a potent inhibitor of C/EBPβ

**Fig 1:**
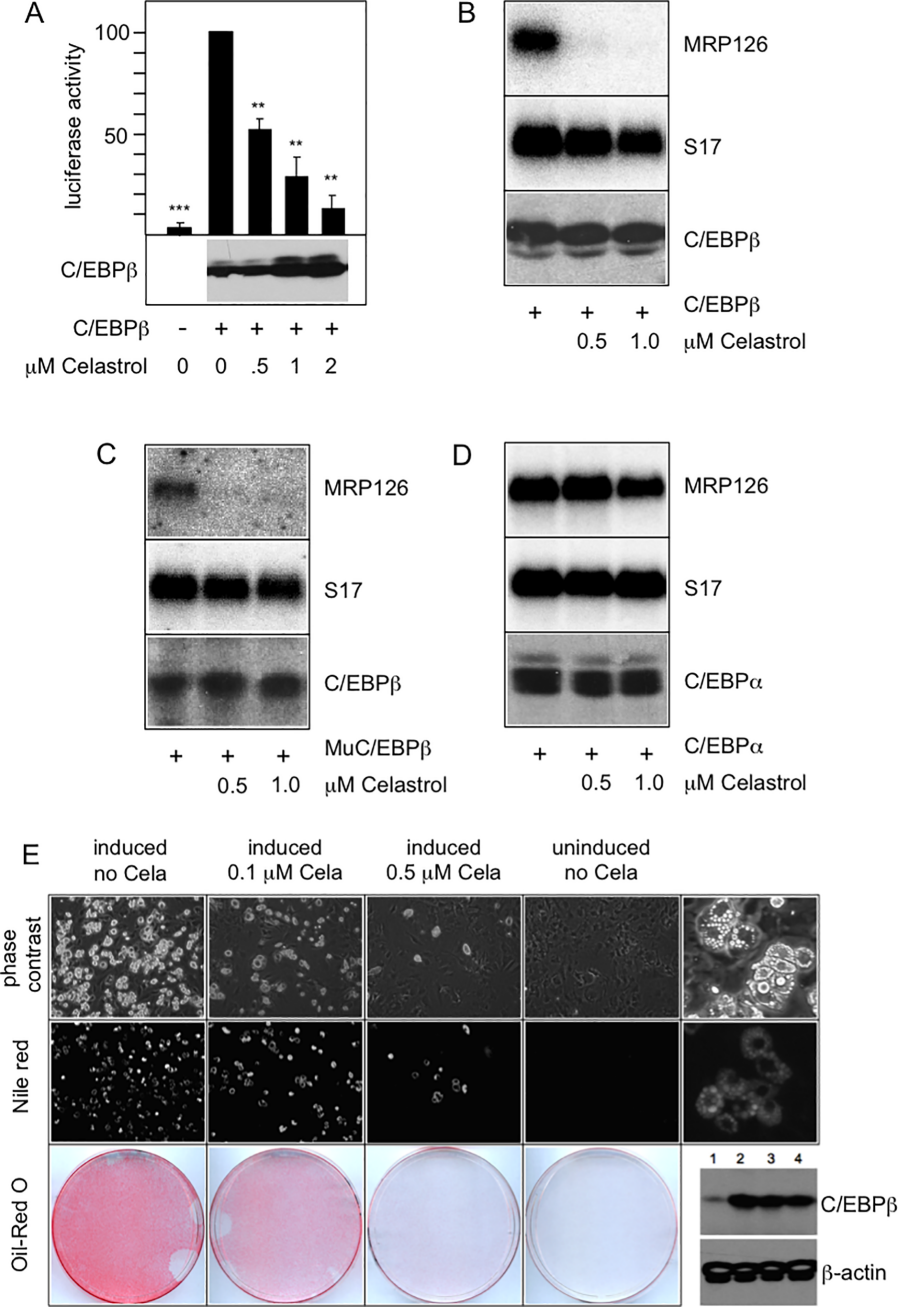
Celastrol inhibits C/EBPβ activity. **A.** QT6 fibroblasts were co-transfected with the luciferase reporter gene p-240luc containing the C/EBP-inducible promoter of the chicken *mim-1* gene, the β-galactosidase expression vector pCMVβ and an expression vector for C/EBPβ transfection, the cells were treated with Celastrol as indicated and harvested after 16 hours. The columns show the average luciferase activity normalized to the β-galactosidase activity. Thin lines show standard deviations. Asterisks indicate statistical significance (** p < 0.01, *** p < 0.001, Student’s t-test). C/EBPβ expression is shown at the bottom. **B-D.** QT6 cells were transfected with expression vectors for chicken or mouse full-length C/EBPβ or chicken C/EBPα and treated with Celastrol as indicated. Cells were harvested after 16 hours and analyzed by northern blotting for expression of *MRP126* and *S17* mRNAs (top and middle panel) and by western blotting for the expression of C/EBPβ or C/EBPα (bottom panels). **E.** 3T3-L1 cells were induced to undergo adipocyte differentiation for 7 days in the absence or presence of the indicated concentrations of Celastrol. The top and middle panels show microscopic pictures of the cells at low magnification and after staining with the fluorescent lipid dye Nile Red. The bottom panels show staining of the cells with Oil-Red O. The top and middle panels at the right show microscopic pictures of differentiated cells at higher magnification. The bottom panels on the right side show western blots of C/EBPβ and β-actin expression in undifferentiated (lane 1) and differentiated (lanes 2–4) cells treated without Celastrol (lane 2) or with 0.5 μM (lane 3) and 1 μM (lane 4) Celastrol.

To investigate if Celastrol previously identified as a Myb inhibitor also affects the activity of C/EBPβ we performed luciferase assays with a reporter construct that is driven the promoter of the *mim-1* gene, which contains high affinity C/EBP binding sites and is strongly activated by C/EBPβ [36]. These experiments clearly demonstrated that Celastrol strongly inhibits the activity of C/EBPβ (Fig 1A). The amount of C/EBPβ was not decreased by Celastrol, indicating that the activity and not the expression of C/EBPβ was inhibited. Importantly, because the reporter assays were performed in fibroblasts that do not express Myb, the inhibition of C/EBPβ activity by Celastrol is independent of its previously described Myb-inhibitory activity [25]. Hence, we concluded that Celastrol inhibits both Myb and C/EBPβ independently of each other.

To demonstrate under more physiological conditions that Celastrol inhibits C/EBPβ, we analyzed its effect on the transcriptional activation of an endogenous C/EBP target gene that is silent in fibroblasts but can be activated by ectopic expression of C/EBPβ or C/EBPα [15,33], thereby providing a read-out of C/EBP activity at a chromatin-embedded gene. Fig 1B shows that Celastrol strongly inhibited the stimulation of MRP126 expression by C/EBPβ, confirming that Celastrol is a potent inhibitor of C/EBPβ. Mouse (Fig 1C) and human C/EBPβ were also strongly inhibited by Celastrol. Interestingly, while Celastrol erased C/EBPβ activity virtually completely at the concentrations tested the related transcription factor C/EBPα was inhibited only slightly under the same conditions (Fig 1D).

To demonstrate the inhibitory activity of Celastrol on C/EBPβ in a different biological context we employed the mouse pre-adipocyte cell-line 3T3-L1, whose differentiation into adipocytes is an established model of a C/EBPβ-dependent differentiation process. In the 3T3-L1 cells, the initial activation of C/EBPβ induces a cascade of gene activations ultimately leading to a fat cell phenotype that is characterized by an accumulation of lipid droplets in the cytoplasm of the cells [32]. Because C/EBPβ plays a key role during the initial phase of adipocyte differentiation we expected Celastrol to inhibit the differentiation of these cells. Fig 1E shows that the massive differentiation observed in the absence of Celastrol was strongly suppressed by Celastrol as demonstrated by microscopic analysis and staining with the lipid dyes Oil Red O and Nile Red. Consistent with previous work [32], as shown by the western blots at the bottom right side of Fig 1E, the amount of C/EBPβ increased when differentiation was induced, but was not significantly diminished by Celastrol, demonstrating that Celastrol inhibits the activity and not the expression of C/EBPβ. Together, the data in Fig 1 show that Celastrol is a very potent inhibitor of C/EBPβ.

### Celastrol disturbs the interaction of C/EBPβ and p300

**Fig 2:**
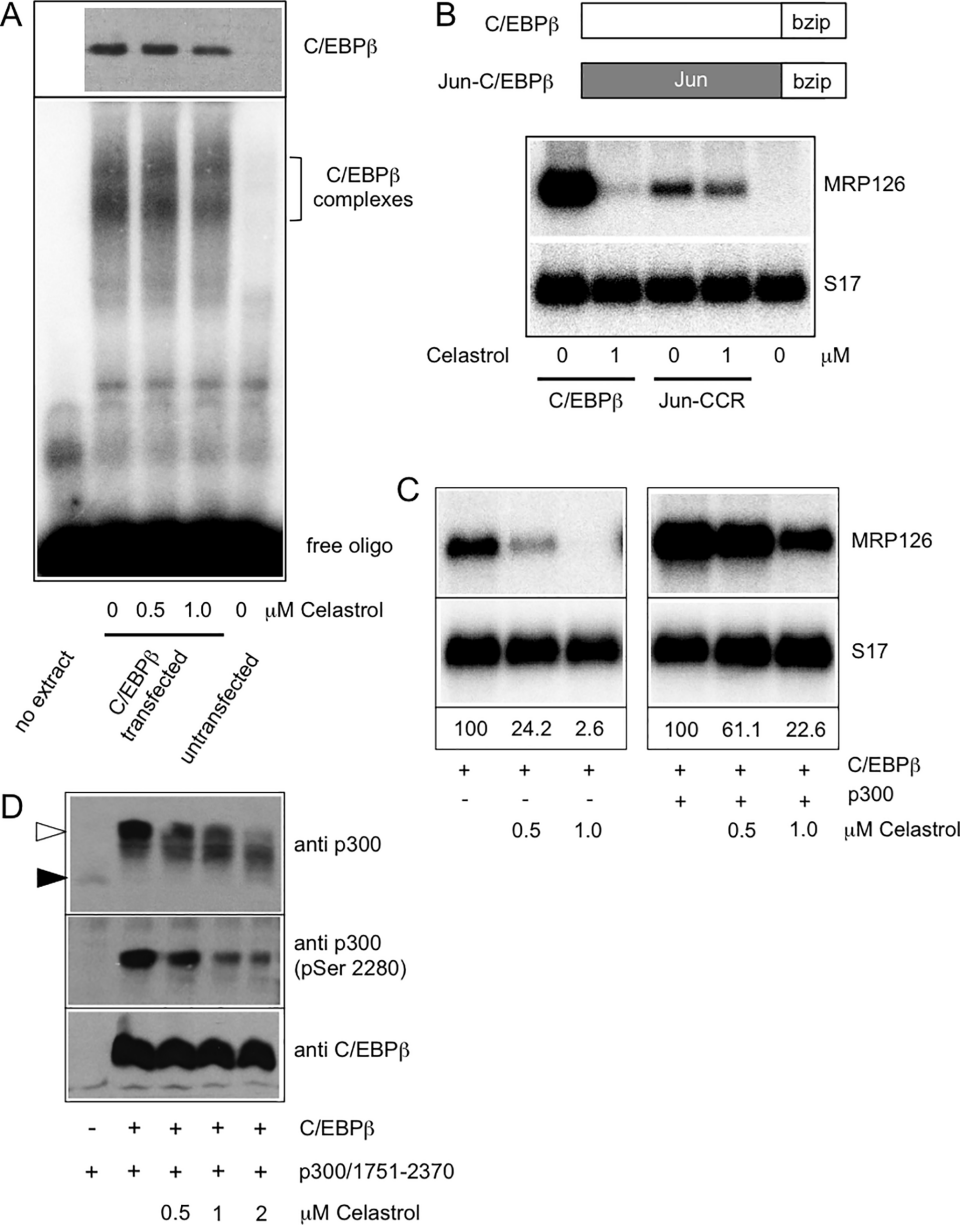
Celastrol does not affect DNA-binding of C/EBPβ but disrupts its cooperation with p300. **A.** A radiolabeled double-stranded oligonucleotide with a consensus C/EBP binding site was incubated without nuclear extract, with nuclear extract from cells transfected with C/EBPβ expression vector and treated with Celastrol as indicated, or with nuclear extract from untransfected cells. Protein-DNA complexes were analyzed by native polyacrylamide gel electrophoresis. The top panel shows a western blot analysis of aliquots of the nuclear extracts stained with antibodies against C/EBPβ. **B.** QT6 cells transfected with expression vectors for C/EBPβ or a Jun-C/EBPβ hybrid protein and incubated with Celastrol were analyzed by northern blotting for expression of *MRP126* and *S17* mRNAs. **C.** QT6 cells transfected with expression vectors for C/EBPβ and p300 were treated with the indicated concentrations of Celastrol, harvested after 16 hours and analyzed by northern blotting for expression of *MRP126* and *S17* mRNAs. Numbers below the lanes indicate the amounts of *MRP126* mRNA relative to the S17 mRNA as control determined by quantification with a phosphor image analyzer. The signals for *MRP126* and *S17* mRNAs were obtained by sequential hybridization of the same blot with specific radiolabeled probes. **D.** QT6 cells transfected with expression vectors for C/EBPβ and p300/1751-2379 and cultivated for 24 hours with or without Celastrol. Aliquots of total cell extracts were then analyzed with antibodies against p300, pp300(Ser2280) and C/EBPβ. Black and white arrows mark the un-phosphorylated and highly phosphorylated p300.

To investigate if Celastrol inhibits C/EBPβ DNA-binding activity we performed electrophoretic mobility shift assays (EMSA) with nuclear extracts from cells transfected with C/EBPβ expression vector and treated with or without Celastrol. Fig 2A shows that the DNA-binding activity of C/EBPβ was not significantly affected by Celastrol. As can be seen in the lower part of Fig 2A, complexes of C/EBPβ bound to the radiolabeled oligonucleotide were not significantly reduced by the treatment of the cells with Celastrol. Similarly, the amount of C/EBPβ present in the nuclear extract was not significantly affected by the Celastrol treatment (see western blot at the top of Fig 2A). That Celastrol did not inhibit C/EBPβ activity by interfering with the function of its DNA-binding domain was confirmed by comparing the effect of Celastrol on the expression of MRP126 mRNA induced by C/EBPβ or a Jun-C/EBPβ hybrid protein containing the DNA-binding domain of C/EBPβ and the transactivation domain of c-Jun (Fig 2B). While Celastrol strongly inhibited the activity of C/EBPβ that of Jun-C/EBPβ was virtually unaffected, clearly indicating that Celastrol does not exert its inhibitory effect via the DNA-binding domain of C/EBPβ. It is also obvious that the activity of C/EBPβ strongly exceeded that of Jun-C/EBPβ (compare first and third lane of Fig 2B). This might be related to the ability of C/EBPβ to function as a pioneer transcription factor [34].

Our previous work has implicated the co-activator p300 as a key cooperation and interaction partner of C/EBPβ [33]. Hence, we were interested to know if Celastrol disrupts the cooperation of C/EBPβ with p300. We first examined if increased expression of p300 overrides the inhibitory effect of Celastrol. We co-transfected fibroblasts with expression vectors for C/EBPβ and p300 and analyzed the effect of Celastrol on the expression of MRP126 mRNA. As control, the cells were transfected only with C/EBPβ expression vector. Fig 2C shows that the inhibition of MRP126 expression by Celastrol was less strong when C/EBPβ was co-expressed with p300 than when it was expressed alone, however, p300 only partially rescued C/EBPβ activity.

**Fig 3:**
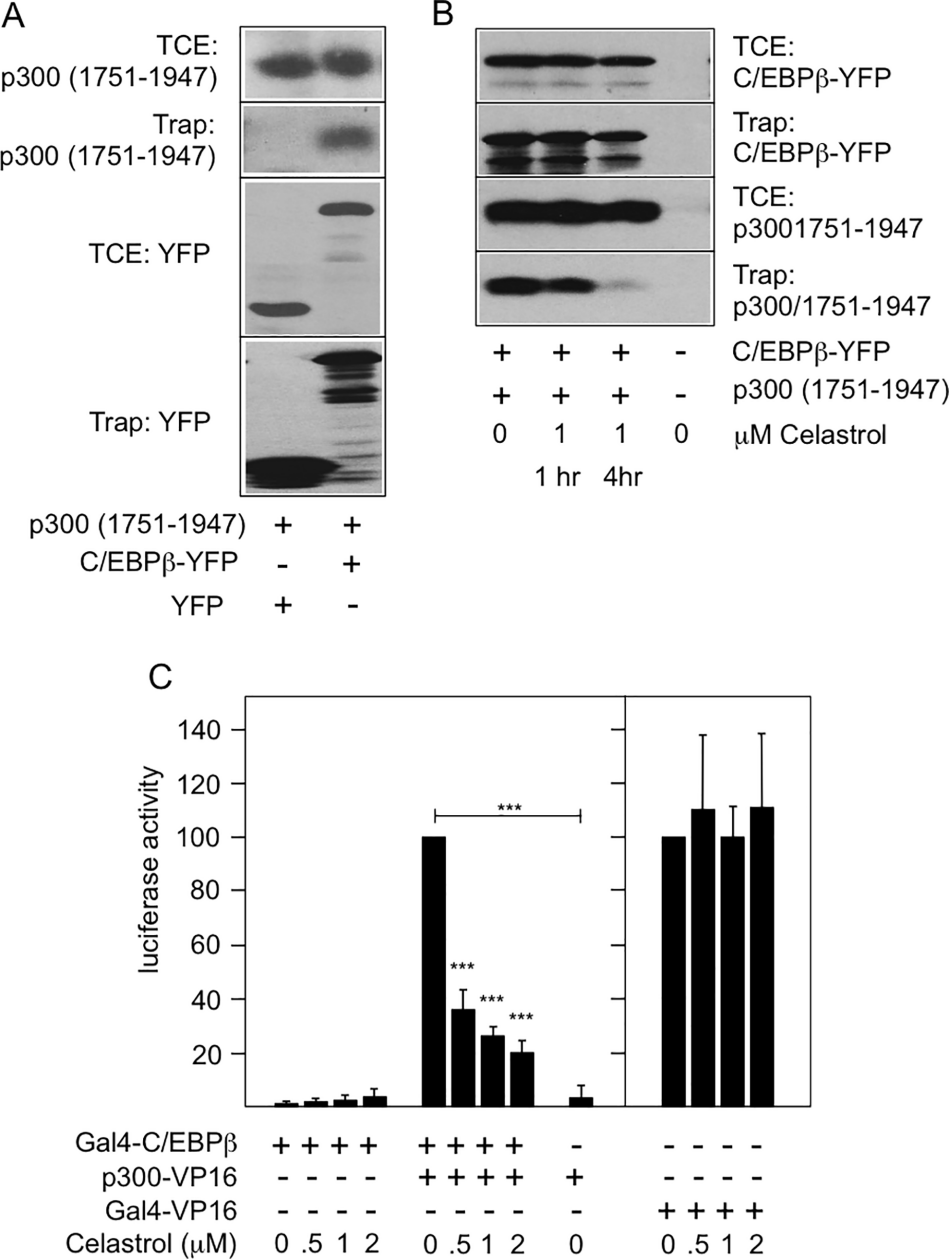
Celastrol disrupts the C/EBPβ-p300 interaction. **A,B.** QT6 fibroblasts transfected with the indicated expression vectors were subjected to GFP-trap experiments. Total cell extracts (TCE) and GFP-trap samples (trap) were analyzed by western blotting with antibodies against GFP and p300. In panel B, the cells were also treated with Celastrol. **C.** QT6 fibroblasts were transfected with the Gal4-dependent reporter gene pG5E4-38luc, the β-galactosidase plasmid pCMVβ and expression vectors for Gal4-C/EBPβ and p300-VP19, as indicated. The transfected cells were incubated for 12 hours with or without Celastrol followed by analysis of luciferase activities. The luciferase activity was first normalized to the β-galactosidase activity. The normalized luciferase activity of the Gal4-C/EBPβ plus p300-VP16 transfected cells or the Gal4-VP16 transfected cells in the absence of Celastrol was then set to 100%. Asterisks indicate statistical significance (*** p < 0.001, Student’s t-test).

We have previously shown that the interaction of p300 and C/EBPβ triggers phosphorylation of multiple sites in the C-terminal domain of p300 thereby increasing its co-activator activity [37]. The C/EBPβ-induced phosphorylation is catalyzed by protein kinase Hipk2, which itself interacts with the amino terminal domain of C/EBPβ [38]. To confirm that Celastrol disturbs the cooperation of C/EBPβ and p300 we analyzed its effect on the C/EBPβ-induced phosphorylation of p300, which can be monitored by the mobility shift of the C-terminal domain of p300 induced by C/EBPβ [37]. We also used an antiserum against phosphorylated Ser-2280 of p300, one of the sites whose phosphorylation is increased by C/EBPβ [38]. Fig 2D shows that Celastrol indeed suppressed the C/EBPβ-induced phosphorylation of p300, as evidenced by the less-pronounced C/EBPβ-induced mobility shift and a decrease in the phosphorylation of Ser-2280. Because co-immunoprecipitation experiments showed that Celastrol did not interfere with the interaction of C/EBPβ and Hipk2 we wondered whether Celastrol disrupts the interaction of p300 and C/EBPβ. To address this we co-transfected fibroblasts with expression vectors for YFP-tagged C/EBPβ and p300/1751-1947, which harbors the C/EBPβ binding region of p300 [33]. We then incubated extracts of the transfected cells with GFP-trap beads (which carry a covalently bound high-affinity GFP binding protein) to precipitate YFP-C/EBPβ together with the bound p300/1751-1947, allowing us to monitor the interaction of both proteins. Fig 3A shows that p300/1751-1947 was co-precipitated with YFP-C/EBPβ whereas expression of YFP as control did not result in co-precipitation, indicating that the interaction between p300/1751-1947 and YFP-C/EBPβ observed in this assay is specific. We then performed co-precipitation experiments with extracts from cells that had been treated with Celastrol for different times before harvesting. Fig 3B shows that Celastrol clearly inhibited the co-precipitation of both proteins, demonstrating that it disrupts the C/EBPβ-p300 interaction.

To substantiate this conclusion we performed a mammalian two-hybrid experiment, using a Gal4-inducible luciferase reporter construct and expression vectors for Gal4-C/EBPβ and a p300/VP16 construct that contains the Taz2 domain of p300 [33,37]. As expected, p300/VP16 strongly stimulated the activity of the Gal4-dependent reporter gene in the presence but not in the absence of Gal4-C/EBPβ (Fig 3C). Increasing concentrations of Celastol gradually reduced the activity of the reporter gene, confirming that Celastrol interferes with the C/EBPβ-Taz2 interaction. Control transfections showed that Celastrol did not affect the activity of Gal4-VP16. This indicated that Celastrol does not inhibit the Gal4 DNA-binding domain or the VP16 transactivation domain. In summary, our data suggest that Celastrol inhibits the activity of C/EBPβ by disrupting the interaction of C/EBPβ with the Taz2 domain of p300.

### Celastrol inhibits the p300-C/EBPβ interaction by targeting cysteine residues located in the Taz2 domain of p300

**Fig 4:**
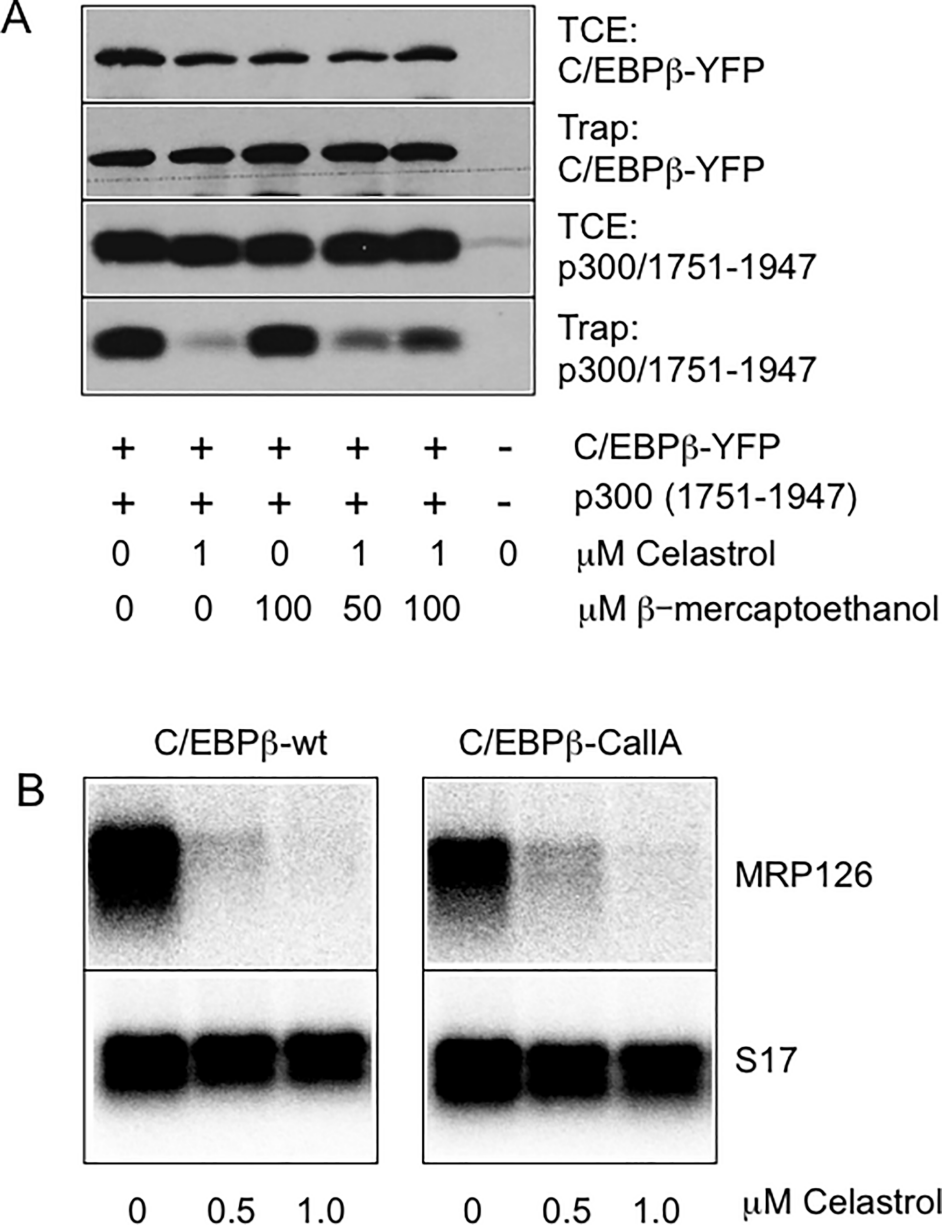
Inhibition of a cysteine-free mutant of C/EBPβ by Celastrol. **A.** QT6 fibroblasts were transfected with the indicated expression vectors and treated with Celastrol and β-mercaptoethanol. Cell extracts were then subjected to GFP-trap and analyzed as in Fig 3. **B.** QT6 cells were transfected with expression vectors for wild-type or cysteine-free C/EBPβ and treated with or without Celastrol. Cells were analyzed by northern blotting for expression of *MRP126* and *S17* mRNAs as in Fig 1 and Fig 2.

Celastrol contains reactive groups that can covalently react with cysteine residues in proteins [39]. To investigate if Celastrol inhibits the C/EBPβ-p300 interaction by alkylating cysteines we first performed GFP-trap experiments in the presence of the thiol reagent β-mercaptoethanol. Cells were transfected with expression vectors for YFP-C/EBPβ and p300/1751-1947, treated with or without β-mercaptoethanol and Celastrol before cell extracts were subjected to co-precipitation analysis. Fig 4A shows that β-mercaptoethanol partially relieved the inhibitory effect of Celastrol, suggesting that alkylation of cysteine residues might be involved in the inhibition of the C/EBPβ-Taz2 interaction by Celastrol. However, this experiment did not reveal whether Celastrol targets cysteine residues of C/EBPβ or p300 (or both). To address this we first used a cysteine-free mutant of C/EBPβ which was still active as determined by its ability to stimulate the expression of the endogenous MRP126 gene. Importantly, the cysteine-free version of C/EBPβ largely retained its sensitivity towards Celastrol (Fig 4B), indicating that Celastrol does not inhibit C/EBPβ by alkylating cysteine residues of C/EBPβ.

**Fig 5:**
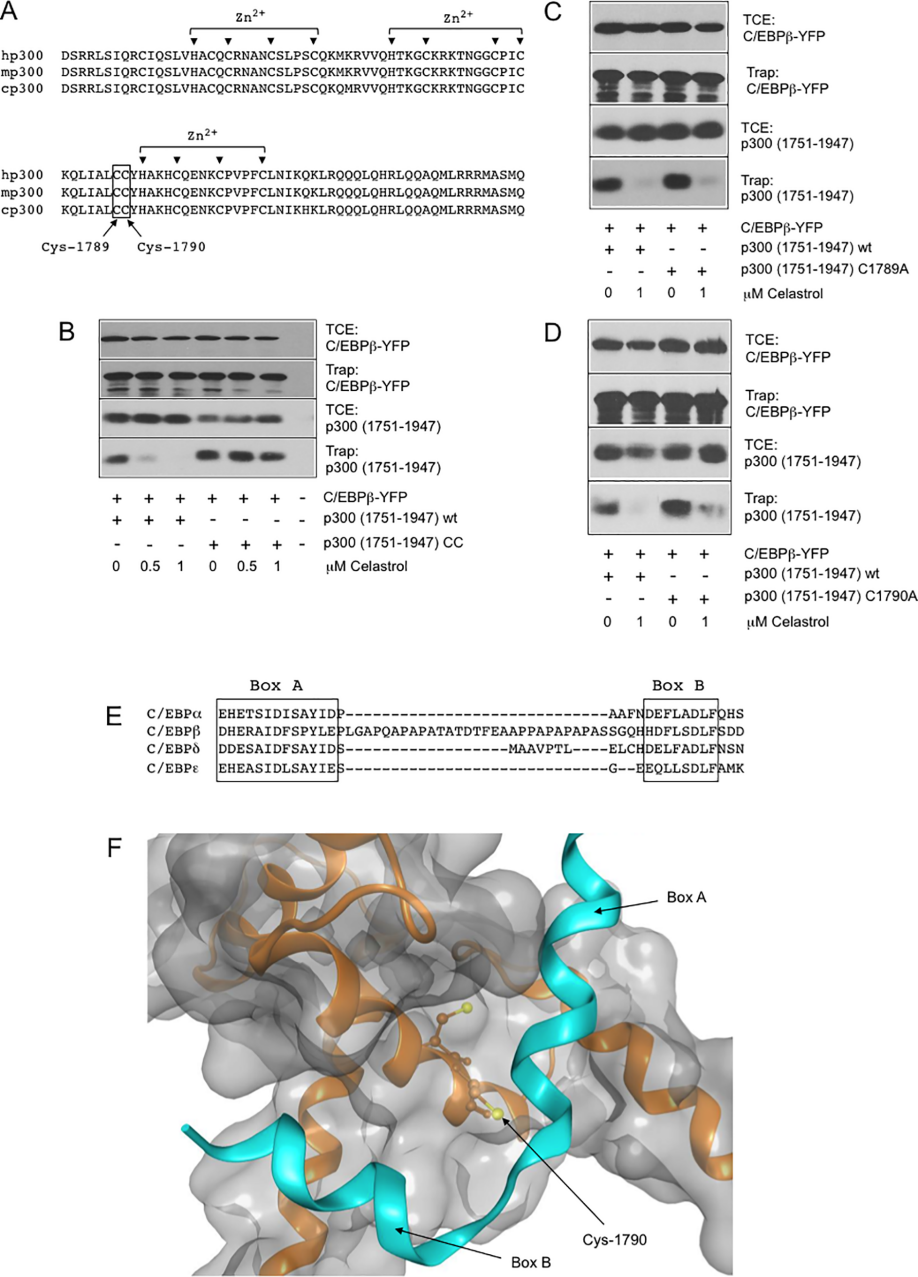
Disruption of the C/EBPβ-p300 interaction is dependent on cysteine residues in the Taz2 domain of p300. **A.** Sequence comparison of the p300 Taz2 domain from human, mouse and chicken. Cysteine and histidine residues involved in coordination of zinc ions and Cys-1789 and Cys-1790 are highlighted. **B-D.** QT6 fibroblasts transfected with the indicated expression vectors were subjected to GFP-trap experiments and analyzed as in Fig 3. **E.** Amino acid sequences of different C/EBP family members implicated in Taz2-binding. Box A and Box B refer to α-helical regions assumed to directly interact with the Taz2 domain. **F.** Partial view of the Taz2 domain and its interaction with Box A and Box B sequences of C/EBPε. The peptide backbones of the Taz2 domain and of CEBPε are shown in brown and turquois, respectively. The position of Cys-1790 is shown. The figure was created from PDB entry 3t92.

The Taz2 domain of p300 also contains cysteine residues, most of which are involved in the coordination of zinc ions to stabilize the three-dimensional structure of the domain [40] (Fig 5A). Because the coordination of zinc ions reduces the nucleophilicity of these cysteine residues they are less likely to react with electrophiles and were not considered as likely targets for Celastrol. However, the Taz2 domain contains two cysteines located immediately adjacent to each other (Cys-1789 and Cys-1790) that are not involved in zinc coordination and, thus, might be potential targets of alkylation by Celastrol. Previous studies have established that C/EBP family members have at least two binding regions (Box A and B, Fig 5E) that mediate direct protein-protein-interactions with the Taz2 domain. According to a recent model based on the interaction of C/EBPε with the Taz2 domain [41] Cys-1789 and Cys-1790 of p300 are located close to the binding region on the Taz2 surface (Fig 5F). To investigate if Cys-1789 and Cys-1790 are involved in the disruption of the C/EBPβ-Taz2 interaction by Celastrol, we mutated them to alanine and examined the ability of the resulting protein to interact with C/EBPβ. As shown by the GFP-trap experiment illustrated in Fig 5B, the C1789/1790A mutant protein was able to interact with C/EBPβ, indicating that the amino acid replacement did not result in massive structural changes of the Taz2 domain. Importantly, the ability of Celastrol to disrupt the C/EBPβ-Taz2 interaction was completely abolished by the mutation. This strongly suggests that one or both of these cysteines are indeed the targets of Celastrol. To investigate if the replacement of a single cysteine residue is sufficient to abolish the inhibitory effect of Celastrol, we also generated mutants in which only Cys-1789 or Cys-1790 were replaced by alanine. Fig 5C and 5D show that the interaction of C/EBPβ with both mutant proteins was still inhibited by Celastrol. Thus, the presence of either Cys-1789 or Cys-1790 is sufficient for the disruption of the C/EBPβ-Taz2 interaction by Celastrol.

## Discussion

We have demonstrated that Celastrol, a natural compound previously shown to inhibit the transcriptional activity of Myb, is also a very potent inhibitor of C/EBPβ. The activity of C/EBPβ is strongly dependent on p300, which is recruited to C/EBPβ via direct protein-protein-interactions between C/EBPβ and the Taz2 domain of p300 [33]. Our data show that Celastrol disrupts the C/EBPβ-Taz2 interaction, most likely by a mechanism that involves the alkylation of cysteine residues by Celastrol. This is supported by the observation that the thiol reagent β-mercaptoethanol reduces the inhibitory effect of Celastrol on the C/EBPβ-Taz2 interaction. We showed that a mutant of C/EBPβ devoid of cysteine residues was inhibited by Celastrol as strongly as wild-type C/EBPβ whereas alanine-substitution of p300 Cys-1789 and Cys-1790, two cysteine residues within the Taz2 domain that are not involved in binding zinc ions, abolished the inhibitory effect of Celastrol on the C/EBPβ-Taz2 interaction. Overall, these findings strongly suggest that Celastrol inhibits C/EBPβ activity by alkylating p300 Cys-1789 or Cys-1790 (or both) and thereby prevents the C/EBPβ-p300 interaction. We have also tried to detect covalent adducts of Celastrol with these cysteines by mass spectrometry. However, because of difficulties in detecting the relevant peptides these experiments were not conclusive. Definitive proof for the suggested mechanism is therefore awaiting more sophisticated mechanistic analyses.

According to a model of the interaction of C/EBPε with the Taz2 domain [41] binding is mediated by a bi-partite binding site, which is conserved among C/EBP family members and located at amino acids 68–80 and 115–123 of C/EBPβ. As shown in Fig 5F, the binding region forms two α-helices that are separated by a turn (or additional sequences in case of C/EBPβ) and interact with the Taz2 domain in the vicinity of Cys-1789 and Cys-1790. It is therefore conceivable that the presence of a bulky ligand, such as Celastrol, at one or both of the cysteines compromises the C/EBPβ-Taz2 interaction. The activity of C/EBPα was only weakly inhibited by Celastrol (Fig 1D), indicating that Celastrol shows a preference for inhibition of C/EBPβ versus C/EBPα. This is surprising because C/EBPα is also known to recruit p300 as co-activator [16,42]. However, the presumed p300 binding regions of C/EBPα and C/EBPβ are not identical; in particular, a sequence stretch that separates the bi-partite p300-binding site of C/EBPβ, is missing in C/EBPα (Fig 5E). This could lead to differences in the binding modes of C/EBPβ and C/EBPα that might explain the differential effects of Celastrol on the activities of both C/EBP family members. Structural studies of complexes of the Taz2 domain with C/EBPβ and C/EBPα are currently not available and will be required to address these issues in future studies. In more general terms, the differential effects of Celastrol on C/EBPβ and C/EBPα clearly illustrate that a compound that acts through reactive groups can nevertheless have quite specific inhibitory activity.

**Fig 6:**
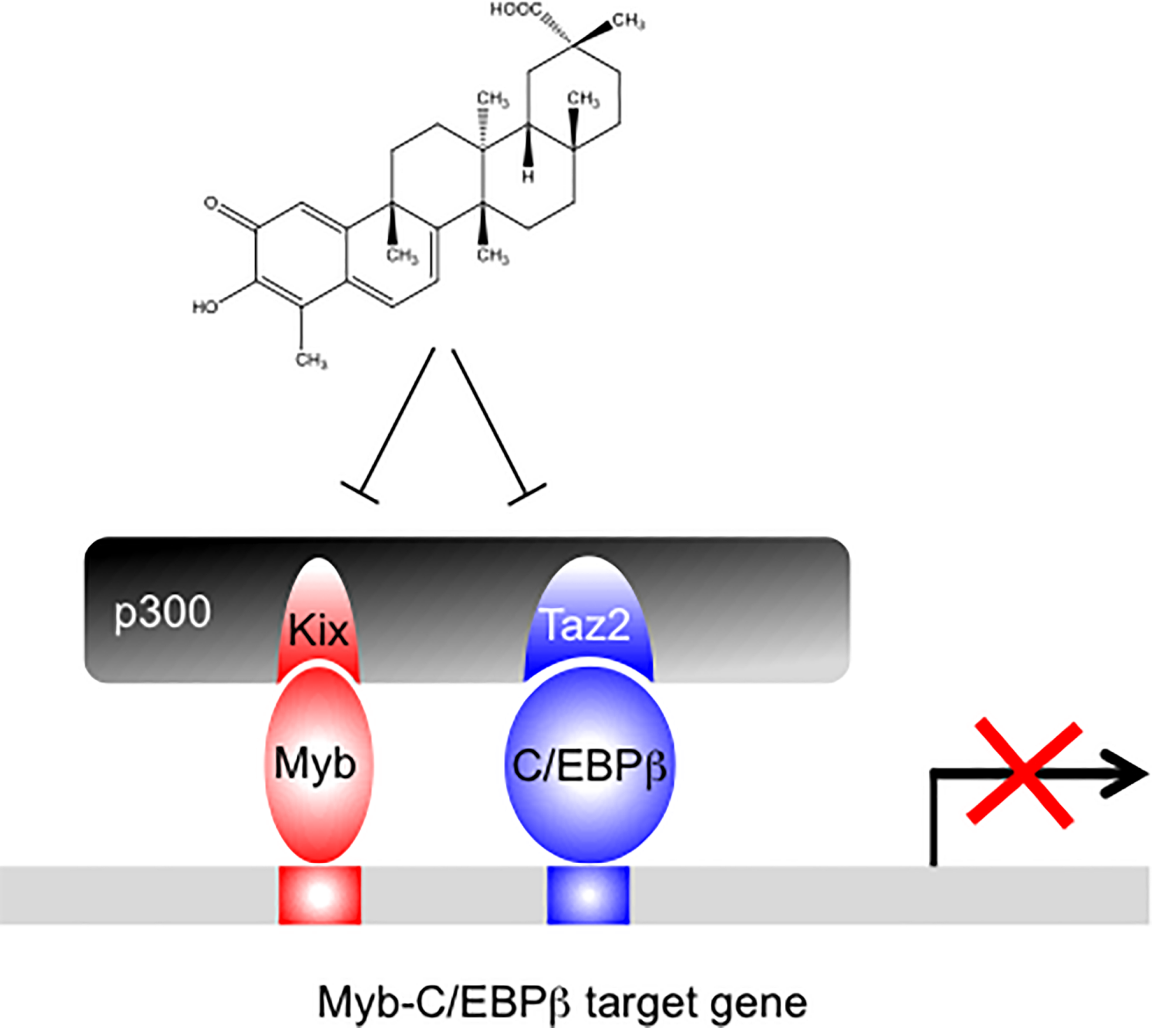
Model of the inhibition of a transcriptional Myb-C/EBPβ-p300 module by Celastrol.

We recently identified Celastrol as a Myb inhibitor using a reporter gene driven by the cis-regulatory elements of the Myb target gene *mim-1* [25]. Mechanistic studies have shown that Celastrol inhibits Myb activity by disrupting the Myb-Kix interaction, thereby depriving Myb of its essential co-activator [25]. The new data presented here provide a more complex view of the inhibitory potential of Celastrol. It appears that the strong inhibitory effect of Celastrol in our screening system was due to the simultaneous suppression of the activities of Myb and C/EBPβ, both of which cooperate tightly at the cis-regulatory elements of the *mim-1* gene. In both cases affects Celastrol the cooperation with the co-activator p300, which independently interacts with Myb and C/EBPβ via the Kix and Taz2 domains, as illustrated schematically in Fig 6. Although this might appear surprising at first glance, such a two-tiered inhibitory mechanism can be rationalized because the activation of the *mim-1* gene by Myb is strongly dependent on the cooperation of Myb with C/EBPβ [30,31]. Furthermore, p300 not only stimulates the activity of Myb and C/EBPβ individually but also enhances their synergistic behavior [33], suggesting that Myb, C/EBPβ and p300 constitute a transcriptional module in myeloid cells. In retrospect, it is therefore not too surprising that a compound that was selected for its ability to strongly inhibit the stimulation of the *mim-1* gene by Myb actually inhibits this transcriptional module by two independent mechanisms. We have recently identified the natural compound Withaferin A as another highly active inhibitor of Myb-induced *mim-1* expression. Interestingly, the analysis of the inhibitory mechanism of Withaferin A has shown that it also acts as a dual Myb and C/EBPβ inhibitor [27].

We have previously shown that Celastrol inhibits the proliferation of leukemic cells from AML patients while not affecting the proliferation of normal hematopoietic progenitors. Furthermore, Celastrol prolonged survival in an in vivo mouse model of an aggressive AML [25]. Recent genome-wide studies have demonstrated that Myb, C/EBPβ and p300 together with several other hematopoietic transcription factors and the bromo-domain protein BRD4 co-localize at many genomic sites in AML cells to stimulate the activity of so-called “super-enhancers” that are involved in maintaining the proliferation of the leukemic cells [11]. Although we currently have no evidence that Celastrol affects the activity of super enhancers in AML cells it is nevertheless conceivable that the inhibition of the Myb-C/EBPβ-p300 transcriptional module by Celastrol might diminish the activity of such enhancers, similar to its effect on the *mim-1* enhancer. Thus, our findings suggest the interesting idea that the promising anti-leukemic effects of Celastrol that we have previously described [25] might be due to the abrogation of Myb, C/EBPβ and p300 function at super-enhancers in AML cells. While this appears to be an interesting possibility, further work is clearly required to validate this notion. It should also be kept in mind that Celastrol is an electrophile that can undergo covalent alkylation reactions with cysteine groups in proteins and thereby might affect a panel of “unspecific” targets. Whether or not Celastrol is an interesting lead molecule for further development of therapeutics against leukemia therefore remains to be seen.

Apart from AML, C/EBPβ shows pro-oncogenic activities in other tumors, such as tumors of the colon, prostate, ovaries, kidneys and the stomach, where C/EBPβ expression often correlates with increased malignancy and invasive activity of the tumor cells [43–45]. A strong case has been made for glioblastoma, where high C/EBPβ expression levels correlate with a poor prognosis for the patient and C/EBPβ together with STAT3 plays a key role in establishing a mesenchymal gene expression signature that is responsible for the aggressiveness of high-grade glioblastomas [46–48]. In the hematopoietic system C/EBPβ plays pro-oncogenic roles in multiple myeloma [49] and anaplastic large cell lymphoma (ALCL) [50]. Overall, C/EBPβ therefore appears to be also an interesting drug target in its own right.
